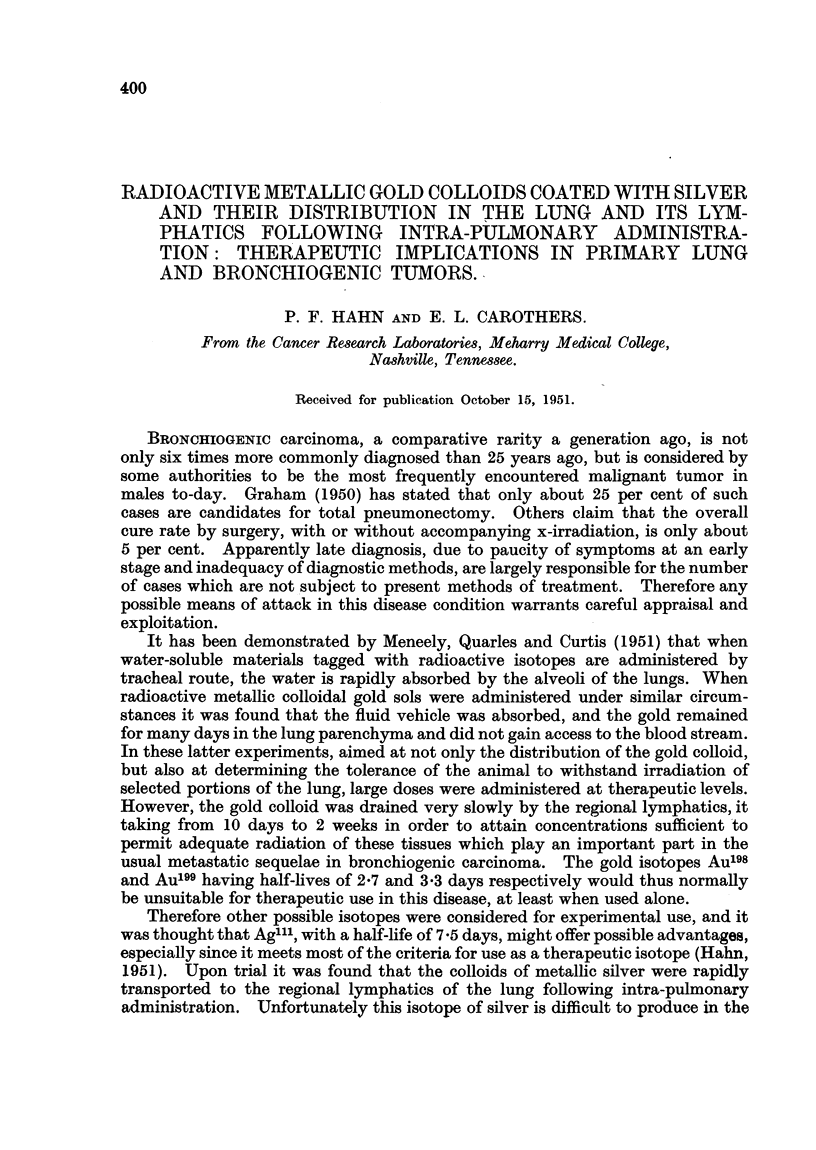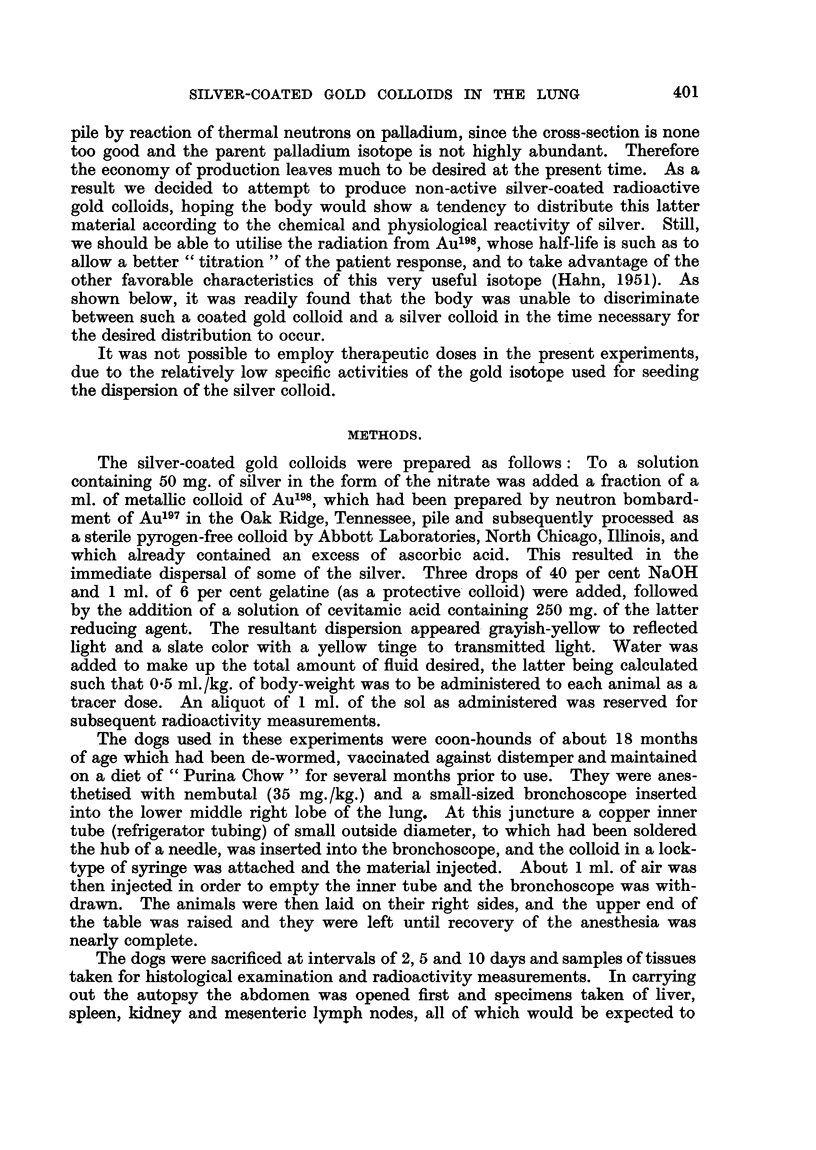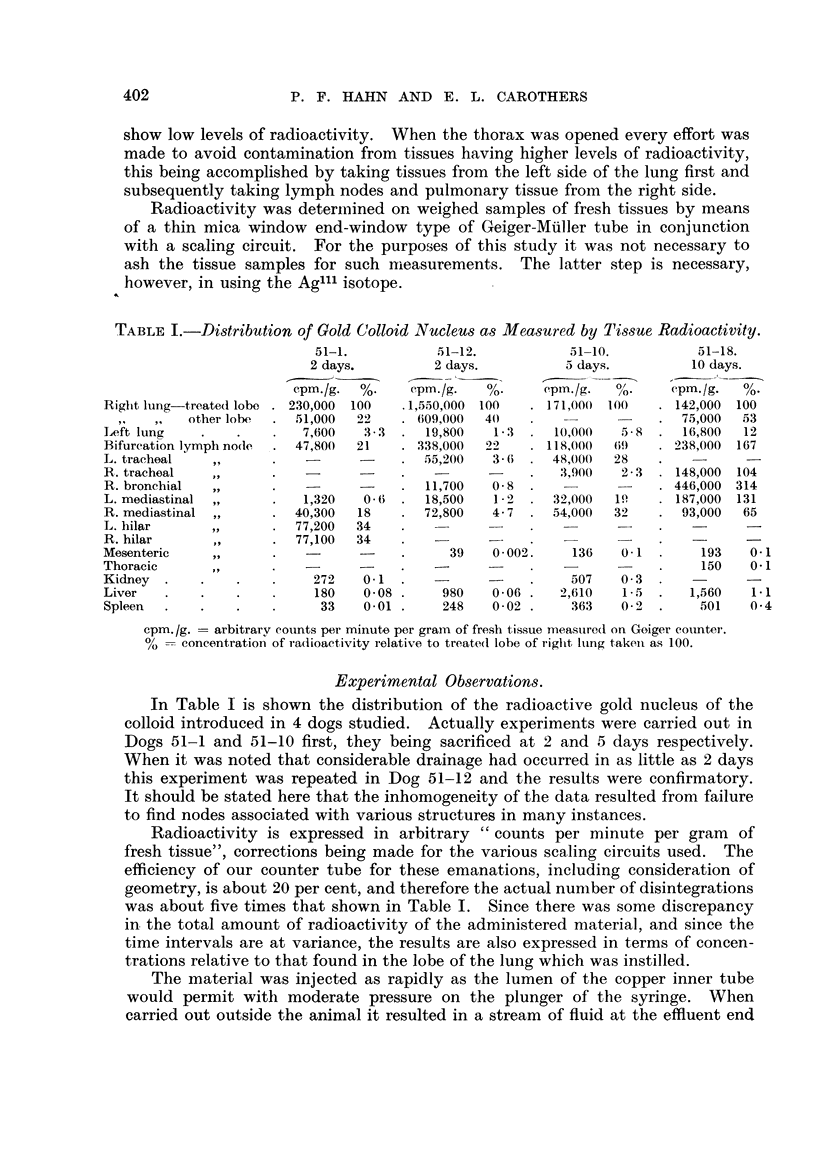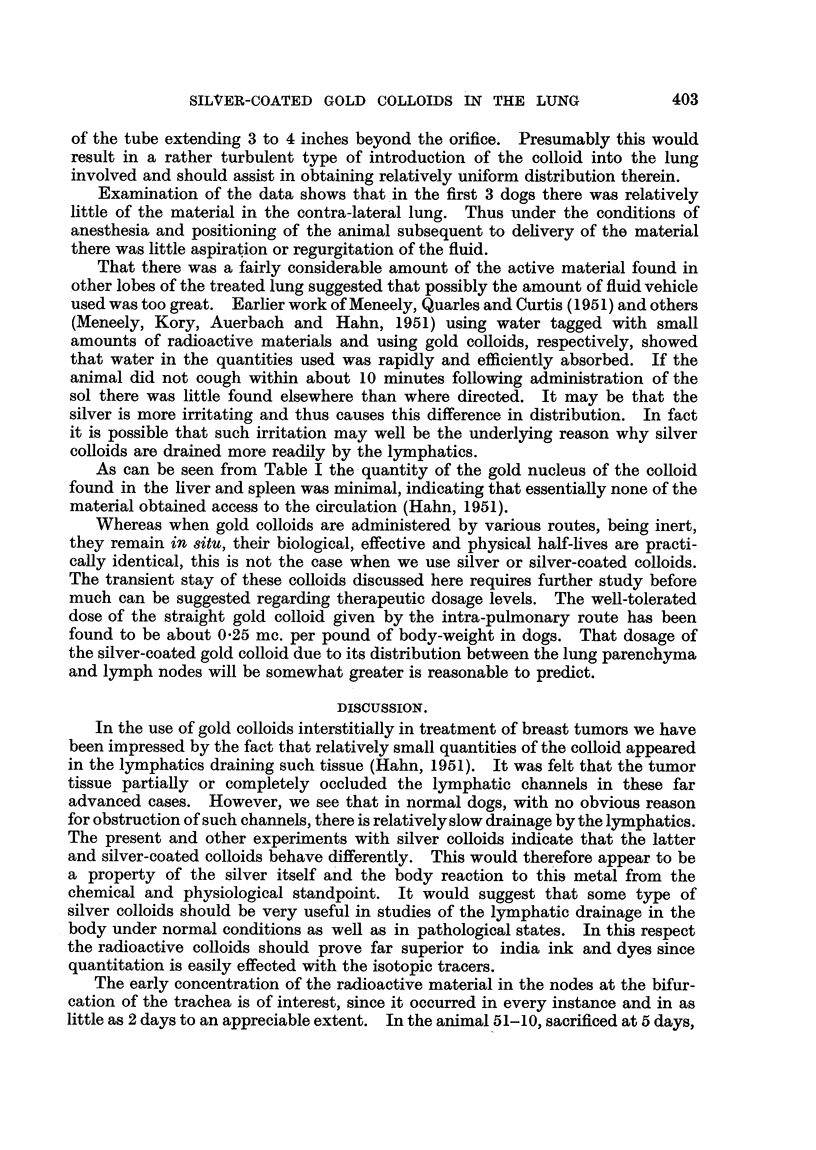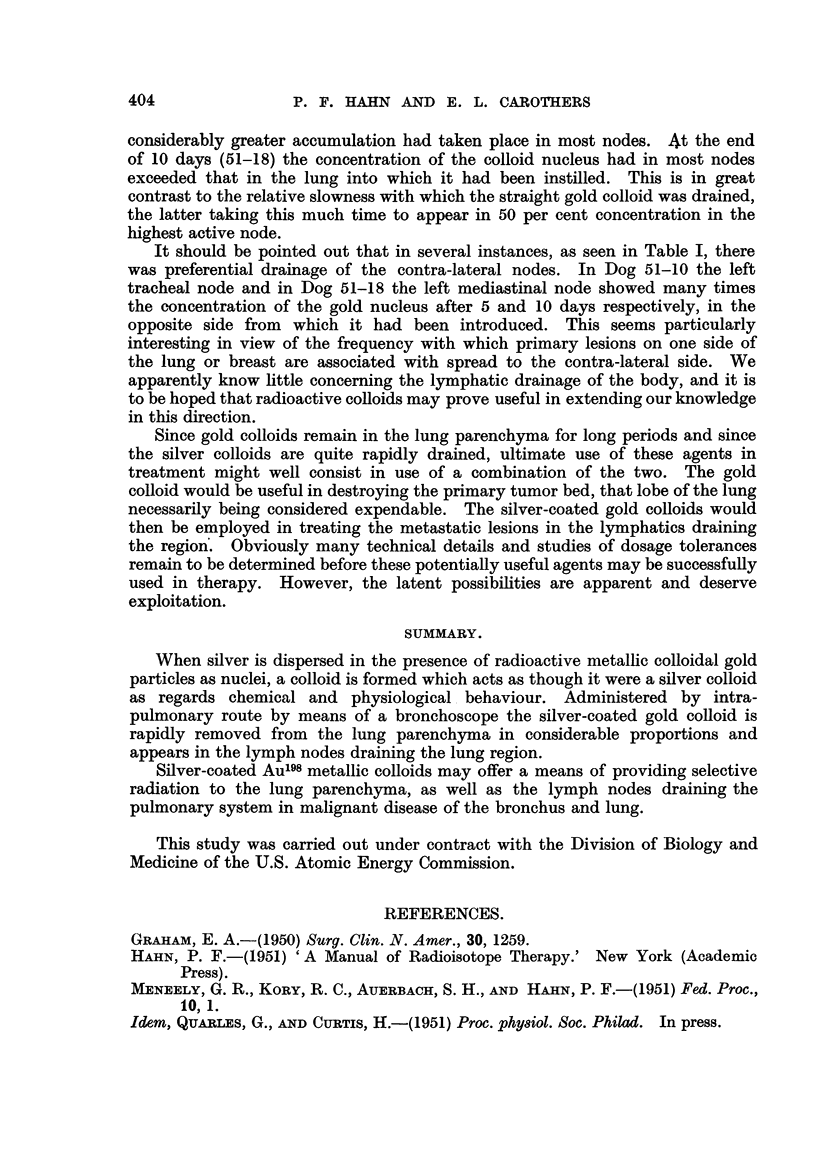# Radioactive Metallic Gold Colloids Coated with Silver and their Distribution in the Lung and its Lymphatics following Intra-Pulmonary Administration: Therapeutic Implications in Primary Lung and Bronchiogenic Tumors

**DOI:** 10.1038/bjc.1951.45

**Published:** 1951-12

**Authors:** P. F. Hahn, E. L. Carothers


### RADIOACTIVE METALLIC GOLD COLLOIDS COATED WITH SILVER AND THEIR DISTRIBUTION IN THE LUNG AND ITS LYMPHATICS FOLLOWING INTRA-PULMONARY ADMINISTRATION: THERAPEUTIC IMPLICATIONS IN PRIMARY LUNG AND BRONCHIOGENIC TUMORS.

P. F. HAHN AND E. L. CAROTHERS.

*From the Cancer Research Laboratories, Meharry Medical College, Nashville, Tennessee.*

Received for publication October 15, 1951.

BRONCHIOGENIC carcinoma, a comparative rarity a generation ago, is not only six times more commonly diagnosed than 25 years ago, but is considered by some authorities to be the most frequently encountered malignant tumor in males to-day. Graham (1950) has stated that only about 25 per cent of such cases are candidates for total pneumonectomy. Others claim that the overall cure rate by surgery, with or without accompanying x-irradiation, is only about 5 per cent. Apparently late diagnosis, due to paucity of symptoms at an early stage and inadequacy of diagnostic methods, are largely responsible for the number of cases which are not subject to present methods of treatment. Therefore any possible means of attack in this disease condition warrants careful appraisal and exploitation.

It has been demonstrated by Meneely, Quarles and Curtis (1951) that when water-soluble materials tagged with radioactive isotopes are administered by tracheal route, the water is rapidly absorbed by the alveoli of the lungs. When radioactive metallic colloidal gold sols were administered under similar circumstances it was found that the fluid vehicle was absorbed, and the gold remained for many days in the lung parenchyma and did not gain access to the blood stream. In these latter experiments, aimed at not only the distribution of the gold colloid, but also at determining the tolerance of the animal to withstand irradiation of selected portions of the lung, large doses were administered at therapeutic levels. However, the gold colloid was drained very slowly by the regional lymphatics, it taking from 10 days to 2 weeks in order to attain concentrations sufficient to permit adequate radiation of these tissues which play an important part in the usual metastatic sequelae in bronchiogenic carcinoma. The gold isotopes $Au^{198}$ and $Au^{199}$ having half-lives of 2·7 and 3·3 days respectively would thus normally be unsuitable for therapeutic use in this disease, at least when used alone.

Therefore other possible isotopes were considered for experimental use, and it was thought that $Ag^{111}$, with a half-life of 7·5 days, might offer possible advantages, especially since it meets most of the criteria for use as a therapeutic isotope (Hahn, 1951). Upon trial it was found that the colloids of metallic silver were rapidly transported to the regional lymphatics of the lung following intra-pulmonary administration. Unfortunately this isotope of silver is difficult to produce in the pile by reaction of thermal neutrons on palladium, since the cross-section is none too good and the parent palladium isotope is not highly abundant. Therefore the economy of production leaves much to be desired at the present time. As a result we decided to attempt to produce non-active silver-coated radioactive gold colloids, hoping the body would show a tendency to distribute this latter material according to the chemical and physiological reactivity of silver. Still, we should be able to utilise the radiation from $Au^{198}$, whose half-life is such as to allow a better " titration " of the patient response, and to take advantage of the other favorable characteristics of this very useful isotope (Hahn, 1951). As shown below, it was readily found that the body was unable to discriminate between such a coated gold colloid and a silver colloid in the time necessary for the desired distribution to occur.

It was not possible to employ therapeutic doses in the present experiments, due to the relatively low specific activities of the gold isotope used for seeding the dispersion of the silver colloid.

### METHODS.

The silver-coated gold colloids were prepared as follows: To a solution containing 50 mg. of silver in the form of the nitrate was added a fraction of a ml. of metallic colloid of $Au^{198}$, which had been prepared by neutron bombardment of $Au^{197}$ in the Oak Ridge, Tennessee, pile and subsequently processed as a sterile pyrogen-free colloid by Abbott Laboratories, North Chicago, Illinois, and which already contained an excess of ascorbic acid. This resulted in the immediate dispersal of some of the silver. Three drops of 40 per cent NaOH and 1 ml. of 6 per cent gelatine (as a protective colloid) were added, followed by the addition of a solution of cevitamic acid containing 250 mg. of the latter reducing agent. The resultant dispersion appeared grayish-yellow to reflected light and a slate color with a yellow tinge to transmitted light. Water was added to make up the total amount of fluid desired, the latter being calculated such that 0·5 ml./kg. of body-weight was to be administered to each animal as a tracer dose. An aliquot of 1 ml. of the sol as administered was reserved for subsequent radioactivity measurements.

The dogs used in these experiments were coon-hounds of about 18 months of age which had been de-wormed, vaccinated against distemper and maintained on a diet of " Purina Chow " for several months prior to use. They were anesthetised with nembutal (35 mg./kg.) and a small-sized bronchoscope inserted into the lower middle right lobe of the lung. At this juncture a copper inner tube (refrigerator tubing) of small outside diameter, to which had been soldered the hub of a needle, was inserted into the bronchoscope, and the colloid in a lock-type of syringe was attached and the material injected. About 1 ml. of air was then injected in order to empty the inner tube and the bronchoscope was withdrawn. The animals were then laid on their right sides, and the upper end of the table was raised and they were left until recovery of the anesthesia was nearly complete.

The dogs were sacrificed at intervals of 2, 5 and 10 days and samples of tissues taken for histological examination and radioactivity measurements. In carrying out the autopsy the abdomen was opened first and specimens taken of liver, spleen, kidney and mesenteric lymph nodes, all of which would be expected to show low levels of radioactivity. When the thorax was opened every effort was made to avoid contamination from tissues having higher levels of radioactivity, this being accomplished by taking tissues from the left side of the lung first and subsequently taking lymph nodes and pulmonary tissue from the right side.

Radioactivity was determined on weighed samples of fresh tissues by means of a thin mica window end-window type of Geiger-Müller tube in conjunction with a scaling circuit. For the purposes of this study it was not necessary to ash the tissue samples for such measurements. The latter step is necessary, however, in using the $Ag^{111}$ isotope.

TABLE I.—*Distribution of Gold Colloid Nucleus as Measured by Tissue Radioactivity.*

| | 51–1. 2 days. | | 51–12. 2 days. | | 51–10. 5 days. | | 51–18. 10 days. | |
|---|---|---|---|---|---|---|---|---|
| | cpm./g. | %. | cpm./g. | %. | cpm./g. | %. | cpm./g. | %. |
| Right lung—treated lobe | 230,000 | 100 | 1,550,000 | 100 | 171,000 | 100 | 142,000 | 100 |
| ,, ,, other lobe | 51,000 | 22 | 609,000 | 40 | — | — | 75,000 | 53 |
| Left lung | 7,600 | 3·3 | 19,800 | 1·3 | 10,000 | 5·8 | 16,800 | 12 |
| Bifurcation lymph node | 47,800 | 21 | 338,000 | 22 | 118,000 | 69 | 238,000 | 167 |
| L. tracheal ,, | — | — | 55,200 | 3·6 | 48,000 | 28 | — | — |
| R. tracheal ,, | — | — | — | — | 3,900 | 2·3 | 148,000 | 104 |
| R. bronchial ,, | — | — | 11,700 | 0·8 | — | — | 446,000 | 314 |
| L. mediastinal ,, | 1,320 | 0·6 | 18,500 | 1·2 | 32,000 | 19 | 187,000 | 131 |
| R. mediastinal ,, | 40,300 | 18 | 72,800 | 4·7 | 54,000 | 32 | 93,000 | 65 |
| L. hilar ,, | 77,200 | 34 | — | — | — | — | — | — |
| R. hilar ,, | 77,100 | 34 | — | — | — | — | — | — |
| Mesenteric ,, | — | — | 39 | 0·002 | 136 | 0·1 | 193 | 0·1 |
| Thoracic ,, | — | — | — | — | — | — | 150 | 0·1 |
| Kidney | 272 | 0·1 | — | — | 507 | 0·3 | — | — |
| Liver | 180 | 0·08 | 980 | 0·06 | 2,610 | 1·5 | 1,560 | 1·1 |
| Spleen | 33 | 0·01 | 248 | 0·02 | 363 | 0·2 | 501 | 0·4 |

cpm./g. = arbitrary counts per minute per gram of fresh tissue measured on Geiger counter.
% = concentration of radioactivity relative to treated lobe of right lung taken as 100.

### *Experimental Observations.*

In Table I is shown the distribution of the radioactive gold nucleus of the colloid introduced in 4 dogs studied. Actually experiments were carried out in Dogs 51–1 and 51–10 first, they being sacrificed at 2 and 5 days respectively. When it was noted that considerable drainage had occurred in as little as 2 days this experiment was repeated in Dog 51–12 and the results were confirmatory. It should be stated here that the inhomogeneity of the data resulted from failure to find nodes associated with various structures in many instances.

Radioactivity is expressed in arbitrary "counts per minute per gram of fresh tissue", corrections being made for the various scaling circuits used. The efficiency of our counter tube for these emanations, including consideration of geometry, is about 20 per cent, and therefore the actual number of disintegrations was about five times that shown in Table I. Since there was some discrepancy in the total amount of radioactivity of the administered material, and since the time intervals are at variance, the results are also expressed in terms of concentrations relative to that found in the lobe of the lung which was instilled.

The material was injected as rapidly as the lumen of the copper inner tube would permit with moderate pressure on the plunger of the syringe. When carried out outside the animal it resulted in a stream of fluid at the effluent end of the tube extending 3 to 4 inches beyond the orifice. Presumably this would result in a rather turbulent type of introduction of the colloid into the lung involved and should assist in obtaining relatively uniform distribution therein.

Examination of the data shows that in the first 3 dogs there was relatively little of the material in the contra-lateral lung. Thus under the conditions of anesthesia and positioning of the animal subsequent to delivery of the material there was little aspiration or regurgitation of the fluid.

That there was a fairly considerable amount of the active material found in other lobes of the treated lung suggested that possibly the amount of fluid vehicle used was too great. Earlier work of Meneely, Quarles and Curtis (1951) and others (Meneely, Kory, Auerbach and Hahn, 1951) using water tagged with small amounts of radioactive materials and using gold colloids, respectively, showed that water in the quantities used was rapidly and efficiently absorbed. If the animal did not cough within about 10 minutes following administration of the sol there was little found elsewhere than where directed. It may be that the silver is more irritating and thus causes this difference in distribution. In fact it is possible that such irritation may well be the underlying reason why silver colloids are drained more readily by the lymphatics.

As can be seen from Table I the quantity of the gold nucleus of the colloid found in the liver and spleen was minimal, indicating that essentially none of the material obtained access to the circulation (Hahn, 1951).

Whereas when gold colloids are administered by various routes, being inert, they remain *in situ*, their biological, effective and physical half-lives are practically identical, this is not the case when we use silver or silver-coated colloids. The transient stay of these colloids discussed here requires further study before much can be suggested regarding therapeutic dosage levels. The well-tolerated dose of the straight gold colloid given by the intra-pulmonary route has been found to be about 0·25 mc. per pound of body-weight in dogs. That dosage of the silver-coated gold colloid due to its distribution between the lung parenchyma and lymph nodes will be somewhat greater is reasonable to predict.

### DISCUSSION.

In the use of gold colloids interstitially in treatment of breast tumors we have been impressed by the fact that relatively small quantities of the colloid appeared in the lymphatics draining such tissue (Hahn, 1951). It was felt that the tumor tissue partially or completely occluded the lymphatic channels in these far advanced cases. However, we see that in normal dogs, with no obvious reason for obstruction of such channels, there is relatively slow drainage by the lymphatics. The present and other experiments with silver colloids indicate that the latter and silver-coated colloids behave differently. This would therefore appear to be a property of the silver itself and the body reaction to this metal from the chemical and physiological standpoint. It would suggest that some type of silver colloids should be very useful in studies of the lymphatic drainage in the body under normal conditions as well as in pathological states. In this respect the radioactive colloids should prove far superior to india ink and dyes since quantitation is easily effected with the isotopic tracers.

The early concentration of the radioactive material in the nodes at the bifurcation of the trachea is of interest, since it occurred in every instance and in as little as 2 days to an appreciable extent. In the animal 51–10, sacrificed at 5 days, considerably greater accumulation had taken place in most nodes. At the end of 10 days (51–18) the concentration of the colloid nucleus had in most nodes exceeded that in the lung into which it had been instilled. This is in great contrast to the relative slowness with which the straight gold colloid was drained, the latter taking this much time to appear in 50 per cent concentration in the highest active node.

It should be pointed out that in several instances, as seen in Table I, there was preferential drainage of the contra-lateral nodes. In Dog 51–10 the left tracheal node and in Dog 51–18 the left mediastinal node showed many times the concentration of the gold nucleus after 5 and 10 days respectively, in the opposite side from which it had been introduced. This seems particularly interesting in view of the frequency with which primary lesions on one side of the lung or breast are associated with spread to the contra-lateral side. We apparently know little concerning the lymphatic drainage of the body, and it is to be hoped that radioactive colloids may prove useful in extending our knowledge in this direction.

Since gold colloids remain in the lung parenchyma for long periods and since the silver colloids are quite rapidly drained, ultimate use of these agents in treatment might well consist in use of a combination of the two. The gold colloid would be useful in destroying the primary tumor bed, that lobe of the lung necessarily being considered expendable. The silver-coated gold colloids would then be employed in treating the metastatic lesions in the lymphatics draining the region. Obviously many technical details and studies of dosage tolerances remain to be determined before these potentially useful agents may be successfully used in therapy. However, the latent possibilities are apparent and deserve exploitation.

### SUMMARY.

When silver is dispersed in the presence of radioactive metallic colloidal gold particles as nuclei, a colloid is formed which acts as though it were a silver colloid as regards chemical and physiological behaviour. Administered by intra-pulmonary route by means of a bronchoscope the silver-coated gold colloid is rapidly removed from the lung parenchyma in considerable proportions and appears in the lymph nodes draining the lung region.

Silver-coated $Au^{198}$ metallic colloids may offer a means of providing selective radiation to the lung parenchyma, as well as the lymph nodes draining the pulmonary system in malignant disease of the bronchus and lung.

This study was carried out under contract with the Division of Biology and Medicine of the U.S. Atomic Energy Commission.

### REFERENCES.

GRAHAM, E. A.—(1950) *Surg. Clin. N. Amer.*, **30**, 1259.

HAHN, P. F.—(1951) 'A Manual of Radioisotope Therapy.' New York (Academic Press).

MENEELY, G. R., KORY, R. C., AUERBACH, S. H., AND HAHN, P. F.—(1951) *Fed. Proc.*, **10**, 1.

*Idem*, QUARLES, G., AND CURTIS, H.—(1951) *Proc. physiol. Soc. Philad.* In press.